# Supplementary material for: Evaluation of MicroRNAs as Non-Invasive Diagnostic Markers in Urinary Cells from Patients with Suspected Prostate Cancer
Source: Diagnostics (Basel). 2020 Aug 9;10(8):578. doi: 10.3390/diagnostics10080578 (PMC7460346; doi:10.3390/diagnostics10080578)
Supplement: Supplementary file 1 [file diagnostics-10-00578-s001.pdf]

Table S1. Relative expression levels of miRNAs and respective fold changes in urinary sediments from Tf and PCa groups using the sub-cohort of patients with PSA levels ≤ 10 ng/mL (*n* = 34).

miRNA	Median relative expression level (x 10^{-3})		Fold change PCa vs Tf	*p* value
	in Tf	in PCa		
miR-125b	31.97	25.32	0.79	0.845
miR-145	16.23	9.68	0.60	0.183
miR-155	15.59	23.52	1.51	0.891
miR-16	663.17	314.83	0.47	0.017
miR-195	292.72	133.56	0.46	0.045
miR-200c	713.34	741.45	1.04	0.593
miR-205	34.88	42.42	1.22	0.593
miR-21	652.71	357.36	0.55	0.183
miR-218	2.45	1.67	0.68	0.593
miR-26a	315.02	162.06	0.51	0.209
miR-375	55.36	55.83	1.01	0.845
miR-96	0.29	0.08	0.28	0.209

Depicted are the median relative transcript levels of the evaluated miRNAs (normalized to the geometric mean of reference RNAs RNU44 and RNU48) in Tf and PCa groups as well as the fold change in the PCa samples compared to the Tf group. *p* values were calculated by the Mann–Whitney U test and then corrected for multiple comparisons by the Benjamini-Hochberg method. miRNA/miR: microRNA, PCa: prostate cancer, PSA: prostate specific antigen, Tf: tumor-free

Table S2. Assessment of the diagnostic power of PSA, PSAD and miRNA expression levels in urinary sediments by ROC curve analyses using the whole patient cohort (*n* = 50).

Parameter	AUC	95% CI	*p* value
PSA	0.564	0.403-0.726	0.656
PSAD	0.708	0.562-0.853	0.031
miR-125b	0.527	0.362-0.693	0.851
miR-145	0.674	0.519-0.829	0.070
miR-155	0.502	0.338-0.665	0.985
miR-16	0.744	0.599-0.888	0.012
miR-195	0.729	0.587-0.871	0.017
miR-200c	0.546	0.376-0.717	0.794
miR-205	0.526	0.357-0.694	0.851
miR-21	0.628	0.468-0.788	0.217
miR-218	0.532	0.369-0.695	0.851
miR-26a	0.678	0.521-0.834	0.070
miR-375	0.513	0.346-0.680	0.928
miR-96	0.609	0.451-0.767	0.306

p values calculated by the ROC curve analysis were corrected for multiple comparisons by the Benjamini-Hochberg method. 95% CI: 95% confidence interval, AUC: area under the curve, miRNA/miR: microRNA, PSA: prostate specific antigen, PSAD: prostate specific antigen density, ROC: receiver operating characteristic

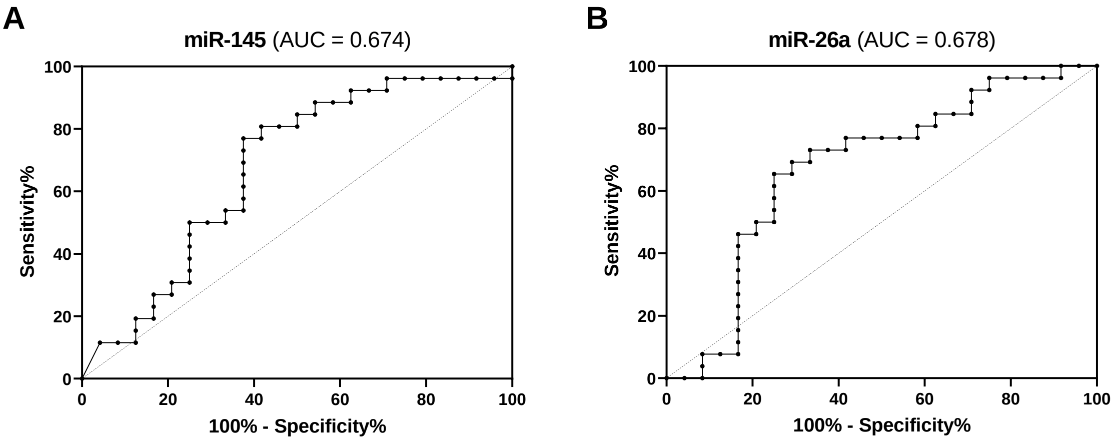

Figure S1. ROC curve analysis of (**A**) miR-145 and (**B**) miR-26a expression levels in urinary sediments using the whole study cohort (*n* = 50). AUC: area under the curve, miR: microRNA, ROC: receiver operating characteristic

Table S3. Assessment of the diagnostic power of PSA, PSAD and miRNA expression levels in urinary sediments by ROC curve analyses using the sub-cohort of patients with PSA levels ≤ 10 ng/mL (*n* = 34).

Parameter	AUC	95% CI	*p* value
PSA	0.621	0.431-0.811	0.379
PSAD	0.595	0.394-0.795	0.524
miR-125b	0.530	0.330-0.730	0.813
miR-145	0.696	0.514-0.879	0.117
miR-155	0.516	0.315-0.716	0.876
miR-16	0.818	0.659-0.976	0.008
miR-195	0.772	0.614-0.930	0.022
miR-200c	0.579	0.384-0.774	0.547
miR-205	0.579	0.380-0.778	0.547
miR-21	0.705	0.522-0.888	0.109
miR-218	0.575	0.380-0.771	0.547
miR-26a	0.674	0.484-0.863	0.155
miR-375	0.530	0.332-0.727	0.813
miR-96	0.674	0.488-0.859	0.155

p values calculated by the ROC curve analysis were corrected for multiple comparisons by the Benjamini-Hochberg method. 95% CI: 95% confidence interval, AUC: area under the curve, miRNA/miR: microRNA, PSA: prostate specific antigen, PSAD: prostate specific antigen density, ROC: receiver operating characteristic

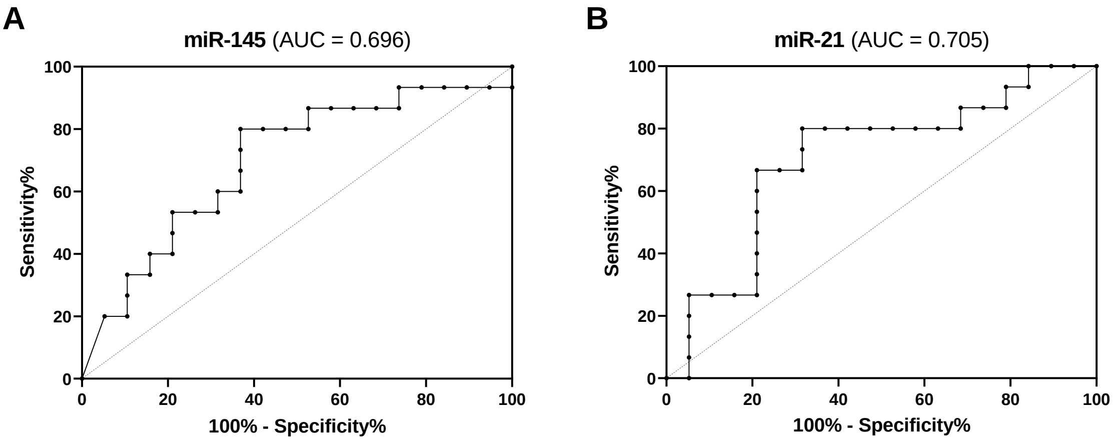

Figure S2. ROC curve analysis of (**A**) miR-145 and (**B**) miR-21 expression levels in urinary sediments using the sub-cohort of patients with PSA levels ≤ 10 ng/mL (*n* = 34). AUC: area under the curve, miR: microRNA, ROC: receiver operating characteristic
